# Case of Dropsy Dependant upon Cirrhosis of the Liver

**Published:** 1842-08-13

**Authors:** 


### CLINICAL REPORTS.

### *Blockley Hospital—Service of W. W. Gerhard, M.D.*

Reported by M. W. Wilson, M. D., Resident Physician.

### *Case of Dropsy dependant upon Cirrhosis of the Liver.*

J. M. G., an Irishman, æt. 29, entered the Hospital on the 26th of March, 1842. He had been previously in the hospital for the same complaint, and a notice of the case was published in a previous No. of the Medical Examiner.

After leaving the hospital he worked on the Lehigh coal wharf for three or four weeks. During that time he remained quite well; he had no dropsy, drank no spirits, and his appetite was good. At this time work declined, and about ten days afterwards he perceived a swelling in his abdomen, with pain in the right hypochondrium. He has had but one chill, which was followed by fever, and preceded the other symptoms. His appetite now became bad, his bowels confined, and his urine scanty and very dark coloured.

March 28th. *Present state.*—Slight jaundice; no œdema of face or arms; abdomen enlarged, and fluctuation can be felt; tympanitis and abdominal tenderness. The scrotum and penis are extremely distended; œdema of extremities moderate. Pulse 68 per minute, small and feeble; circulation feeble through the capillaries; veins distended; tongue slightly coated, and yellowish. The urine is scanty, of a dark yellowish-green colour, thick and muddy in appearance, and exhibits the changes characteristic of the presence of bile by the addition of hydrochloric and nitric acids.

He was treated with purges of jalap and bitartrate of potassa (ten grains of the former, and two drachms of the latter) daily. Occasionally the colchicum mixture, (℞. Vin. Sem. Colchici; Magnesiæ, *aa.* ʒi. Magnes. Sulph. ℥i. Aquæ Simp. ℥iv.) was substituted, when it could be used without producing too much irritation on the stomach and bowels. He also took ten drops of the nitro muriatic acid three times a day; and a bandage was applied to the abdomen, so as to make pressure from above downwards. Two or three days after his entrance a few punctures were made in the scrotum with a lancet, and the fluid allowed to exude. Erysipelas of a pretty severe grade followed the puncturation of the scrotum, and extended up the abdomen, yielding, however, to applications of leadwater and laudanum.—Half an ounce of bitartrate of potassa was also prescribed in juniper tea, which he drank daily.

This treatment was pursued until the 4th of April, with considerable benefit—the jaundice became less marked, and the dropsy diminished. About this time a troublesome cough commenced, with frothy expectoration, dyspnœa, and the physical signs of bronchitis, for which he took an infusion of ipecac. This was followed by a sudden attack of cerebral disturbance, delirium, contraction of the forehead, and distorted expression. When aroused with questions he would answer them correctly, and immediately fall into a stupid or comatose condition again. His pulse at this time was moderately full, and about 80 per minute. A blister to be applied to the nucha, sinapisms to the chest and extremities, and a turpentine injection were prescribed, and followed by great relief. In about forty-eight hours the cerebral symptoms disappeared. The treatment for dropsy—temporarily suspended during the cerebral disturbance—was again pursued, and with much benefit. On the 13th of April his urine was increased to four pints in twenty-four hours, (previously about a pint in twenty-four hours,) and the ascites and œdema greatly diminished. The purges were now discontinued, in consequence of the irritation they were exciting in the stomach and bowels.

From this time the effusion in the abdomen commenced increasing, and became so large, notwithstanding the administration of diuretics and diaphoretics, that pressing against the diaphragm produced dyspnœa so severe as to threaten suffocation. This was always very much increased in the evening, and diminished in the morning. The fear of erysipelas, which several of the patients were labouring under in the hospital at the time, prevented us from resorting to paracentesis abdominis for some time; but the symptoms become so alarming, and the danger of immediate suffocation so great, that it was performed on the 28th of April, and four gallons of fluid taken from him. Symptoms of local peritonitis manifested themselves immediately around the puncture, but a number of leeches, followed by a warm poultice, gave entire relief. His treatment after this was diuretics, diaphoretics, and occasionally purges. The œdema left the extremities, and the abdomen commenced enlarging very much within twenty-four hours, and continued to increase gradually, and his general health to fail, until the 9th of May. He was at this time very prostrate, and had hæmorrhage from the mouth and nose. He was now again seized with a cerebral affection, which increased gradually until the 11th, when he died.

*Necroscopy twenty-four hours after Death.*

The arachnoid membrane was opaque, with patches of bright red, stellated, injection over the upper surface of both hemispheres. The substance of the brain was healthy, and there was no effusion in the ventricles. There was a slight effusion in the pleura; with this exception the viscera of the thorax were nearly healthy.

The peritoneum contained about four gallons of fluid.

The liver was very much atrophied, about half the usual size. In its external appearance it was irregularly knobbed from partial development of the acini. The hypertrophied acini were very large; some were the size of a hazlenut, others smaller. The colour was a light straw yellow.

The mucous membrane of the stomach was of a dark slate colour, and softened throughout.

The intestines exhibited but slight traces of disease.

The kidneys were slightly granulated, apparently the commencement of granular degeneration.

[The patient had been previously cured of the dropsical effusion during a previous residence in the hospital, so that he was able to work for several weeks at a laborious employment, exposed to the dampness of the river. Whether his health would have continued good if he had been able to follow a less injurious avocation is difficult to say. The disease of the liver was beyond remedy; but many patients who labour under these complaints are capable of enjoying life, and do not necessarily relapse into dropsical diseases when once relieved of them; still, they must be placed under circumstances which are not unfavourable, or the ascites will inevitably recur.

The disease of the liver offered a very good illustration of cyrrhosis in an advanced degree, so much so as to have nearly destroyed the proper function of the organ.—W. W. G.]

